# How many people in the world do research and development?

**DOI:** 10.1111/1758-5899.13182

**Published:** 2023-02-08

**Authors:** Davut Emrah Ayan, Laurel L. Haak, Donna K. Ginther

**Affiliations:** ^1^ Institute for Policy & Social Research The University of Kansas Lawrence Kansas USA; ^2^ Ronin Institute and Mighty Red Barn Townsend Wisconsin USA; ^3^ Department of Economics and Institute for Policy & Social Research The University of Kansas Lawrence Kansas USA

## Abstract

The traditional approach to comparing research and development (R&D) capacity across countries has been to compare Gross Domestic R&D expenditures (GERD). In this paper, we argue for an expansion of R&D capacity that includes people engaged in research and research and development activities (research human capital density, RHCD). To achieve this goal, we first discuss how to estimate counts of researchers and create a measure of researcher human capital density within a country. Next, we examine whether RHCD is a useful variable in models of innovation capacity. Finally, we consider whether RHCD has explanatory power for models of research outputs including patents and publications. We find that RHCD has more explanatory power than GERD in the production of patents and publications. We argue that surveys of individuals that include questions on R&D activities are useful for assessing innovation capacity, and, if adopted more broadly, can provide a strategic framework for countries and regions to develop human capital to support innovative activities.

**Policy Implications**

- To align with the more inclusive definition of ‘researcher’ in the 2015 Frascati manual, ‘researcher’ and ‘R&D person’ should be equated in UNESCO data as any person that engages in or provides services to directly support R&D as a primary or secondary activity during their workday.
- Firm‐based (employer) surveys provide insight on R&D activities at the organization, sector, and national level. However, they do not shed light on the occupations or educational background of the people engaged in research activities. We recommend that UNESCO adopt individual and occupational surveys to measure self‐reported research and development work activities to support more inclusive assessment of R&D personnel, education flows, and innovation capacity.
- We encourage countries to augment their industrial reporting with occupational surveys to better understand the relationships between educational investments and workforce. An option is to implement this data collection through census procedures, or through a regular survey of tertiary education completers.
- R&D firms can assist with the determination of human capital density by surveying employees and providing anonymized educational data to national statistical agencies.

## 1 HUMAN CAPITAL AND INNOVATION

Human capital is a key factor in innovation in response to public needs (Belmonte da Silva & Fernandez Jardón, 2021; Blind, 2012; Hamdan & Hamdan, 2020; Lewis et al., 2017). Organizations absorb and utilize knowledge through structural, human, and social capital (Engelman et al., 2017; Soo et al., 2017; Zhu et al., 2020). Firms and universities combine capital and labour to produce innovation. Most policymakers focus on the funding of research & development (R&D) measured by Gross Domestic Expenditures on Research & Development (GERD), whilst largely ignoring labour inputs. This paper considers how research human capital density (RHCD) contributes to innovation on a global scale in comparison to the traditional measure of R&D intensity measured by GERD.

Early quantitative analysis of R&D focused on relationships between research and productivity through new product innovation and production efficiency gains (Ewell, 1955). Innovation indicators are supported by the national collection of economic data, including the Community Innovation Survey (CIS), developed in the early 1990 s, which is a firm‐based survey of innovation inputs and outputs, including investments in innovation, sales of new or changed products, plus data on collaboration, knowledge flows and other topics (Arundel & Smith, 2013). In addition, international economic and education data are collected by OECD, Eurostat, and UNESCO.

Over time, these surveys have evolved, as have the indicators, generally through a bottom‐up consensus process, involving several communities of practice including data producers and analysts, policy analysts and implementers and rule makers (Gault, 2013). The CIS has added and adjusted questions to support analysis and policymaking. Early innovation indicators, such as the ratio of R&D expenditures to sales, are making way for indicators with more nuance (Godin, 2008) that include human capital measures.

Jorgenson and Vu (2013) argue that innovation has a modest role in world economic growth, and instead investments in human capital have a far more important role in both advanced and emerging economies. They point to new statistical datasets, including KLEMS (BEA, 2022) that support the analysis of human capital alongside other components of productivity.

The OECD has been a critical player in innovation policy, collecting and disseminating economic and education data to support the development of science and technology policies in Western countries (Godin, 2004), including methods and standard definitions that enabled comparison across countries, published as the Frascati Manual in 1963 and updated regularly since (OECD, 2015).

Subsequent UNESCO involvement in R&D data gathering and dissemination has helped to enable comparisons across countries on a global scale (UNESCO, 2014). UNESCO adopted the OECD Frascati Manual as the reference tool for their global data collection and supported community work to further harmonize and update definitions between 2010 and 2015. The definition of R&D was updated to: ‘creative and systematic work undertaken to increase the stock of knowledge – including knowledge of humankind, culture and society – and to devise new applications of available knowledge’. (OECD, 2015).

This change, encompassing social sciences and humanities, traditional knowledge, as well as new data coding and collection advice to developing countries, along with increasing attention to human capital in economic growth, compels us to examine how ‘researcher’ is defined. The updated R&D definition exposes assumptions about training, degree, discipline, employment sector, and primary outputs, and opens opportunities for a more holistic and global analysis of innovation capacity (Carayannis et al., 2018; Lau & Lo, 2015; Radziszewski, 2020; Schmidt, 2010) than was afforded by the data prior to 2015.

The U.S. National Science Board's Science & Engineering Indicators (2020) measures R&D intensity as the ratio of GERD (Gross Domestic Expenditures on Research & Development) to GDP (Gross Domestic Product). Whilst R&D personnel figures have been reported for many years (see Jackson, 1966), they focus on the numbers of people and do not encompass the human capital productivity noted above.

UNESCO reports 7.8 million full‐time equivalents (FTE) researchers in 2013 (UNESCO, 2013). This figure represents firm‐reported employment data from G20 countries, only 10% of all countries in the world. With university programs graduating doctorates in over 160 countries and ranked universities in over 100 countries, the UNESCO figure is surely an undercount.

To get at research human capital, we need to know the number of researchers (often measured as R&D personnel) in a place, as well as their educational characteristics. With that information, we can infer the R&D sector capacity in that country and better understand knowledge sharing on a local and global scale, in private, public and government sectors (Kristjánsson et al., 2014; Wagner et al., 2015). In turn, this can provide a strategic framework for nations to develop and support human capital for activities necessary for solving the world's challenging sustainable development goals.

In this paper, we develop a measure of ***research human capital density (RHCD)*** using publicly collected data on R&D personnel. We hypothesize that RHCD will better characterize the innovative capacity of a country because it measures the most critical input in the R&D process—the researchers. We use this measure to address the following research questions: (1) How to estimate the number of researchers in the world; (2) Is research human capital density a useful variable in models of innovation capacity? and (3) Does research human capital density have explanatory power for the research production function of research outputs including patents and publications? Next, we used this measure in cross‐national estimates of publications and patents to determine whether RHCD has sufficient explanatory power. Our results show that once RHCD is included in the research production function, research intensity measured by GERD has limited explanatory power.

## 2 METHODS AND DATA

We had two primary goals in this study. First, we determined the definition and then measured RHCD on a global scale. We started with the UNESCO definition of R&D personnel and then expanded it to include people doing R&D as a primary or secondary job activity using survey data from the United States. Second, we assessed the innovation capacity of countries and regions by examining relationships between researcher counts (FTEs, counts and extrapolations) and innovation inputs (country‐level investments in R&D, educational engagement and attainment), outputs (research papers and patents), as well as the environment (governance metrics and university rankings). We used data from open sources with global reach and intercountry data quality standards and have created a study data set that is available for reuse (Haak et al., 2021).

### 2.1 Sources

**FIGURE 1:**
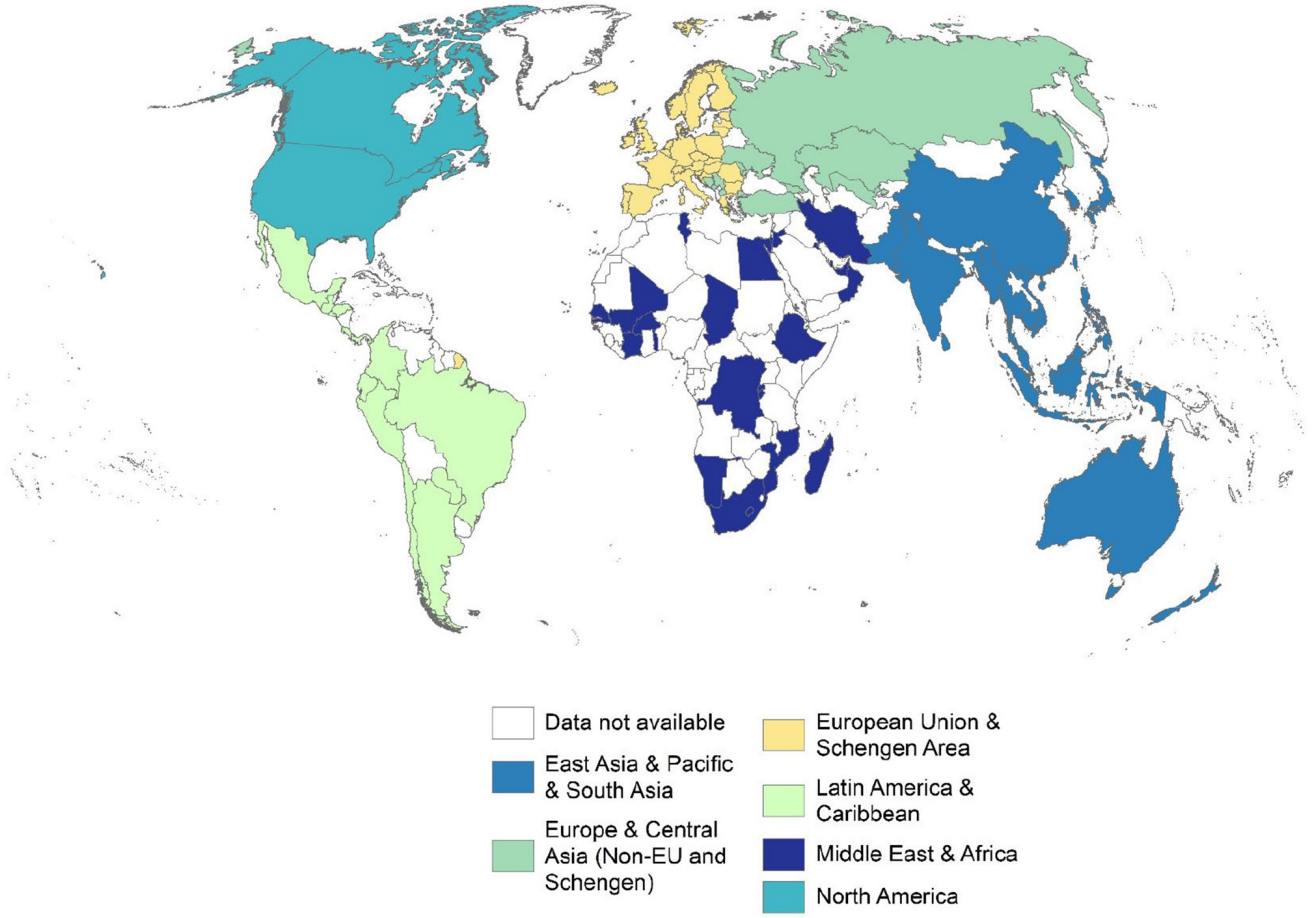
Countries in the final data set shown on a Winkel global projection.

The data used in our analyses were collected from publicly available sources, in most cases with global scope. We obtained data on educational statistics, R&D employment, gross domestic expenditure on R&D (GERD) and general country demographic data (including population) from the World Bank World Development Indicators (WDI, 2021), UNESCO Institute for Statistics (UIS, 2021), and OECD Data (2021) and employment data from the International Labour Organization (ILOSTAT, 2021). We also utilized US‐specific data on the highly educated workforce, namely the US National Science Foundation Survey of Doctoral Recipients (SDR, 2015) and the US Census National Survey of College Graduates (NSCG, 2017). Although data collected may share the same sources, data coverage varies by country (see Table 1 and Figure 1).

**TABLE 1:** Countries in the data set, organized by region.

Region	Countries
APAC: East Asia, Pacific, and South Asia (*n* = 19)	Australia, Brunei Darussalam, Cambodia, China, Hong Kong SAR (China), India, Indonesia, Japan, Macao SAR (China), Malaysia, Myanmar, New Zealand, Pakistan, Philippines, Singapore, South Korea, Sri Lanka, Thailand, Vietnam
ECA: Europe and Central Asia, not EU or Schengen Area (*n* = 11)	Bosnia and Herzegovina, Georgia, Kazakhstan, Moldova, Montenegro, North Macedonia, Russian Federation, Serbia, Turkey, Ukraine, Uzbekistan
EUS: European Union, Schengen Area, and Common Travel Area (*n* = 31)	Austria, Belgium, Bulgaria, Croatia, Cyprus, Czech Republic, Denmark, Estonia, Finland, France, Germany, Greece, Hungary, Iceland, Ireland, Italy, Latvia, Lithuania, Luxembourg, Malta, Netherlands, Norway, Poland, Portugal, Romania, Slovak Republic, Slovenia, Spain, Sweden, Switzerland, United Kingdom
LATAM: Latin America and Caribbean (*n* = 15)	Argentina, Brazil, Chile, Columbia, Costa Rica, Ecuador, El Salvador, Guatemala, Honduras, México, Panamá, Paraguay, Perú, Puerto Rico, Trinidad and Tobago
MEA: Middle East and Africa (n=27)	Bahrain, Burkina Faso, Burundi, Chad, Dem Rep. Congo, Côte d'Ivoire, Egypt, Ethiopia, Iran, Israel, Jordan, Kuwait, Lesotho, Madagascar, Mali, Mauritius, Mozambique, Namibia, Oman, Qatar, Rwanda, Senegal, Seychelles, South Africa, Togo, Tunisia, United Arab Emirates
NA: North America (*n* = 2)	Canada, United States

Downloaded raw data files were cleaned and transformed using Stata/SE version 16.1. Further transformation into data tables and descriptive analysis including graphing and correlation analysis were performed using R version 4.1.0. Multivariable regression analysis was performed using Stata. Maps were created using ESRI's ArcMap (v10.8.1) utilizing country boundaries from ESRI (v10.2, 2015) in the Winkel Tripel projection. Data are grouped into five classes using either the Jenks method or by quintile distribution.

Countries were our unit of analysis, and we used aggregate statistics for the years from 2014 to 2018. Prior to 2014, these data were only available for OECD countries, thus limiting our goal of performing a cross‐country analysis at a global scale. For US‐specific analyses, we used data from the 2015 Survey of Doctoral Recipients (SDR, 2015) which is matched to publications, and the 2017 National Survey of College Graduates (NSCG, 2017). Naturally, countries vary by many dimensions, primarily by population and geography, and these dimensions have consequences for other variables. For that reason, we normalized educational, investment, and employment variables at the country level by dividing them by the population segment aged 25–69.

### 2.2 Data definitions

#### 2.2.1 Regions and countries

Our intent was to maximize the global coverage of our dataset, with a sample size of at least 10 countries in each of the six regions. We examined international country lists provided by OECD, G20, and World Bank, and used World Bank regional groupings as they provided the most complete coverage (World Bank, 2021). From an initial list of 217 countries, we selected those with at least 1 year of data for the period 2014–2018, for the measures of: (a) doctoral education enrollment or attainment; (b) gross domestic expenditure on research and development (GERD); and (c) researcher full‐time equivalents (see ‘Variables’ section below). We expanded the dataset by imputing researcher data when possible. We imputed Australian researcher FTE from Employment in professional, science and technical activities data; Peruvian and Israeli researcher FTE from researcher headcount data (UIS, 2021).

Some countries that have strong tertiary education sectors did not have GERD or researcher data in the UNESCO or World Bank sources during 2014–2018 (or in the 5 years prior) and could not be included. This was a particular challenge in the Middle East and Africa region, affecting Saudi Arabia, Kenya and Nigeria. UNESCO partners with the African Science, Technology and Innovation Indicators (ASTII) Initiative of the African Union to support economic and education statistical data collection. This region is home to 12 countries ranked in the top 25 fragile states (Fund for Peace, 2021), four of which (Chad, Ethiopia, Mali and Mozambique) have sufficient data coverage for inclusion in this study.

Our final data set included 105 countries in five regions spanning the globe (Figure 1 and Table 1).

#### 2.2.2 Variables and coverage

Summary tables of variables and availability for each country, aggregated by region, are provided in Supplementary Tables S1a–e.

##### National investments

Country‐level data on Gross Domestic Product (GDP) (WDI) and Gross Domestic Expenditure on Research and Development (GERD) (UIS) were available for all countries in our dataset. For most countries, we were also able to obtain information on GERD in education and business sectors. All investment data were normalized per capita for the 25–69 aged population subset and log‐transformed prior to running correlations and regressions. Table S1a shows country‐level data availability for the investment variables GDP, GERD total and by sector, aggregated by region. Our measure of GERD is per capita whilst many organizations report GERD as a share of GDP. We use the per capita measure because our other measures are also normalized by population.

##### Educational intensity

As noted above, we selected countries based on the availability of core educational data. We made the assumption that researchers will have completed a college degree. Ideally, we would have examined the full range of educational enrollments and attainments, from a technical degree (International Standard Classification of Education (ISCED) 5) to a doctoral‐level degree (ISCED 8), as well as field of study. However, the data did not support this broad examination for more than OECD countries. ISCED definitions were re‐defined in 2011 and implemented in 2014 (UNESCO, 2014), limiting the year range we could examine. We were able to obtain or impute total tertiary (ISCED 5–8) and doctorate (ISCED 8) enrollment or attainment data for all countries in the dataset (UIS). All educational data were normalized per capita for the age range 25–69 and log‐transformed prior to running correlations and regressions. Table S1b shows country‐level data availability for the educational variables, enrollment and attainment, total tertiary and doctorates, aggregated by region.

##### Researcher counts

**FIGURE 2:**
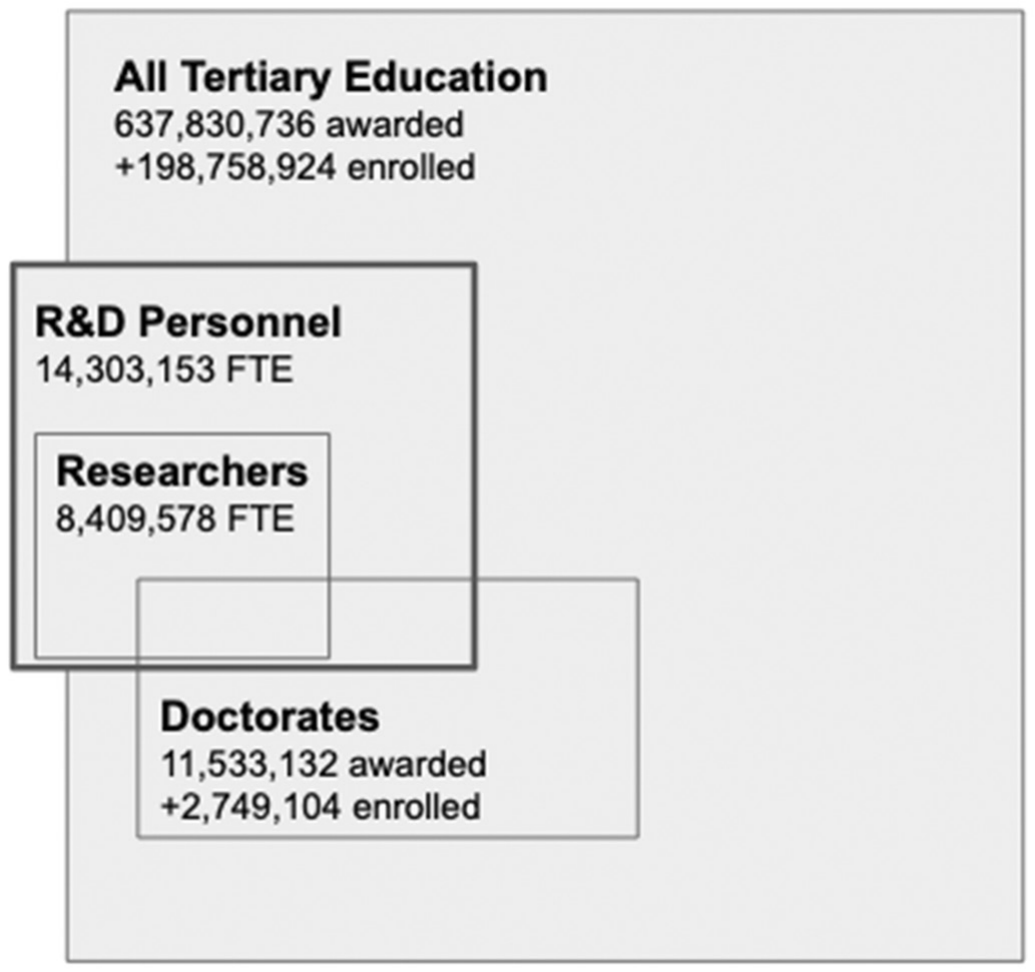
R&D Human Resources, average 2014–2018. *Source:* UNESCO.

The lines between education, research, development, design, and application are difficult to ascertain (see examples and discussion in OECD, 2015). We tested several methods to assess how many people are engaged in R&D activities. Our goal was regional comparisons, so we focused on UNESCO data, but we also extrapolated counts from US individual‐level surveys. Table S1c shows country‐level data availability for employment and researcher‐related variables, aggregated by region. For regressions and correlations, we normalized all data by 1000 per capita for the age range 25–69 and before log transforming. There are two measures: (i) researchers and (ii) R&D personnel, and these measures are reported as ‘head count’ and ‘full‐time equivalents’ with better data coverage in our set for FTEs. R&D personnel are defined in Frascati as all persons engaged directly in R&D including managers, technicians, and administrators. Researchers are a subset of total R&D personnel, and per Frascati include professionals engaged in the conception or creation of new knowledge, conducting research, improving concepts, theories, models, techniques, instrumentation, software, or operational methods (see OECD, 2015). The relationship between researchers and R&D personnel is illustrated in Figure 2.

1. **The total personnel counts (head counts) and FTEs (full‐time equivalents) employed in R&D and as researchers** (UIS). These data are derived from country‐level employer surveys carried out in business, education, government and nonprofit sectors. Notably, whilst business R&D FTE data are available for the US ^1^ total R&D FTE data are missing. For the US we imputed total FTE using sector employment ratios from the NSCG, sponsored by the NSF and carried out every other year by the US Census Bureau (Burke, Finamore, Foley, Jankowski, Moris, 2021). Our analysis is based on FTE throughout the paper.
2. **Self‐reported data on R&D activity from the US.** We were interested in comparing self‐reported and employer‐reported data on R&D activities. Whilst the US does not collect workforce totals from employer surveys, it does field demographic surveys and collects extensive workforce information directly from individuals. These data provide a lens into work activities, occupations, and educational background. We used the NSCG to collect information on survey respondents who reported R&D as a primary and/or secondary activity, as well as educational and occupational variables. We compared these figures with those from employer survey data after normalizing for total tertiary attainment and adjusted this ratio based on country‐level reporting variations for FTE and counts (see below) to estimate R&D personnel counts.
3. **The total number of people who completed a college degree** (ISCED 5–8) (UIS). This provides an estimate of tertiary educational engagement and a broad scope of country‐level reservoir for innovation but may not include people who contribute to R&D through traditional knowledge, self‐taught or trades pathways.
4. **The total number of people who completed a doctoral degree** (ISCED 8) (UIS). This provides a more focused estimate of educational engagement for those people who have engaged in a course of study that encourages novel thinking and research approaches. We extended this analysis using NSF data to explore relationships between doctorates, publications, and grants.

##### Research output measures

We focused on data that were indicative of innovative activity (National Science Board, 2020; OECD/Eurostat, 2018) that were also available for our country data set. We used patenting activity (patent application by residents) from the World Intellectual Property Organization Patent Report (WIPO PatentScope, 2020) and research publication volume from the National Science Board Science and Engineering Indicators (NSB, 2020). Patents provide one measure of industry‐level R&D whilst publications measure university‐level R&D. To mitigate skewing and the adverse effects of outliers, we binned and log‐transformed each variable to obtain a more normal distribution for correlation and regression analyses. We captured the count of ranked universities per country, using the Academic Ranking of World Universities (ARWU, 2019) and CTWS Leiden Rankings (CWTS, 2020) which presents data for the time period 2015–2018; these surveys are largely based on faculty productivity as measured by paper production. We used total counts normalized per million population and binned these data (0, 1–4, 5 or more). Table S1d shows country‐level output data availability for patent applications, publications and ranked universities, aggregated by region.

##### Social factors

In addition to economic and educational factors, we examined the impact of social factors on innovation capacity. For this, we used the World Bank Worldwide Governance Indicators (WGI, 2021) data on government effectiveness, control of corruption, political stability, rule of law, voice and accountability and regulatory quality compiled from over 30 sources reporting the perceptions of governance of many survey respondents and expert assessments. Data were available for all the countries in our dataset worldwide. We used index scores for each country, averaging across the 2014–2018 year range. Table S1e shows country‐level governance environment data availability, aggregated by region.

#### 2.2.3 Research production function estimation

Global comparisons of R&D inputs tend to focus on GERD (National Science Board, 2020). However, both GERD and RHCD are inputs in the knowledge and innovation production processes. The research production function posits that universities and firms combine capital measured by investments in R&D and labour measured by R&D personnel to produce research outputs of publications and patents. This approach has been widely used in the economics literature (see, for example, Rosenbloom et al., 2015). At the country level, we posit that knowledge is a function of:

$$y_{i}=f\left(L_{i},K_{i}\right)$$

where *y*_*i*_ is our measure of patents and publications for country *i*, *L*_*i*_ is R&D human capital (RHCD) and *K*_*i*_ is investment, R&D intensity (GERD). Since this is a cross‐country analysis, knowledge production will also be influenced by educational attainment and research infrastructure measured by the number of ranked universities. In addition, country governance variables may influence how countries convert inputs into knowledge. To operationalize this model, we estimate a with log–log specification using Ordinary Least Squares:

$$\log\left(y_{i}\right)=\alpha +\beta \;\log\left(L_{i}\right)+\delta \;\log\left(K_{i}\right)+\theta \;Z_{i}+\varepsilon_{i}$$

Our analysis uses country‐level data for one period (2014–2018). *Y*_*i*_ is the knowledge outcome in each country (patents, publications), *L*_*i*_ is RHCD, *K*_*i*_ is GERD and *Z*_*i*_ is a matrix of education, governance variables, and region dummies to account for region‐specific characteristics. Coefficients can be interpreted as elasticities and standard errors are robust to heteroskedasticity.

## 3 RESULTS

### 3.1 Summary statistics

Summary tables are presented in Table 2 and include counts, and when applicable, mean, median, and standard deviation for each variable. In the supplement, we probe the distribution of these variables across the region (Table S2a–g). In terms of research output, North America leads the world in patent applications and scientific journal articles. However, East Asia, Pacific and South Asia (APAC) countries have more ranked universities. North America and APAC had 30‐fold higher patent volumes than other regions. The European Union Schengen (EUS) region has a high number of publications but not of patents (Table S2b).

**TABLE 2:** Summary statistics for the study data set.

Variable	N	Mean	Std. Dev.	Min	Pctl. 50	Max
East Asia & Pacific & South Asia	19	18.10%				
Europe & Central Asia (Non‐EU and Schengen)	11	10.50%				
European Union & Schengen Area	31	29.50%				
Latin America & Caribbean	15	14.30%				
Middle East & Africa	27	25.70%				
North America	2	1.90%				
Number of ranked universities	105	11.59	31.75	0	1.00	226.00
Number of Patent applications, residents	91	22,353	124,593	1.75	378	1,122,778
Number of Scientific and technical journal articles	103	22,481	63,950	12.42	2,782	447,684
GDP ‐ per capita	105	48,369	37,738	2,431	43,088	188,064
GERD ‐ per capita	105	503.10	700.61	0.50	167.39	2,782.64
GERD Business Sector ‐ per capita	85	379.07	539.07	0.05	120.34	2,411.37
GERD Higher Education Sector ‐ per capita	102	133.29	173.38	0.13	51.86	705.10
R&D personnel FTE ‐ per 1000 population	105	5.07	5.43	0.05	2.64	24.45
Researcher FTE ‐ per 1000 population	105	3.40	3.72	0.03	1.62	15.58
Employment ‐ per capita	104	0.83	0.18	0.49	0.82	1.37
Tertiary attainment, total ‐ per 1000 population	102	251.28	149.81	8.39	224.96	775.30
Tertiary attainment, Doctorate ‐ per 1000 population	102	4.96	5.73	0.11	2.58	35.50
Tertiary enrollment, total ‐ per 1000 population	103	60.49	29.43	9.98	58.88	155.53
Tertiary enrollment, Doctorate ‐ per 1000 population	103	1.34	1.35	0.01	0.93	6.08
Government Effectiveness	105	0.41	0.92	(1.58)	0.24	2.22
Control of Corruption	105	0.28	0.99	(1.40)	0.02	2.25
Political stability and Absence of Violence	105	0.06	0.88	(2.41)	0.13	1.53
Regulatory Quality	105	0.45	0.92	(1.46)	0.42	2.18
Rule of Law	105	0.33	0.96	(1.62)	0.20	2.05
Average score on "Voice and Accountability"	105	0.20	0.93	(1.83)	0.24	1.69

Research funding also varies by region. North America has double the GERD per capita compared to EUS and three times the GERD per capita compared to APAC countries (Table S2c). North America leads the world in tertiary educational attainment per 1000 population at 548, followed by Europe and Central Asia (ECA) at 341 and EUS at 338. North America has double the number of doctorates per capita compared to EUS and over 4 times the number of APAC (Table S2f).

### 3.2 Counting researchers: Research human capital density

#### 3.2.1 Global data: UNESCO Employer surveys

We started the counting process by examining UNESCO's UIS R&D personnel data. These data are collected using an annual employer questionnaire (UIS, 2022) based on definitions of R&D and personnel encoded in the Frascati Manual (OECD, 2015):

- R&D is defined to ‘comprise creative and systematic work undertaken in order to increase the stock of knowledge – including knowledge of humankind, culture and society – and to devise new applications of available knowledge’. R&D includes basic and applied research and experimental development.
- R&D personnel includes all persons engaged directly in R&D, whether directly employed or external contributors, as well as those providing direct services for the R&D activities such as R&D managers, administrators, researchers, technicians and clerical staff. R&D personnel perform scientific and technical work for an R&D project (setting up and carrying out experiments or surveys, building prototypes, etc.); plan and manage R&D projects; prepare interim and final reports for R&D projects; provide internal services for R&D projects (e.g. dedicated computing or library and documentation work), and provide support for the administration of the financial and personnel aspects of R&D projects.
- R&D personnel excludes individuals undertaking indirect support or ancillary activities in R&D‐performing units, such as specific services to R&D provided by central computer departments and libraries, services by central finance and personnel departments dealing with R&D projects and R&D personnel, and the provision of services for security, cleaning, maintenance, canteens, etc., to R&D‐performing units.
- Researchers are defined as professionals engaged in the conception or creation of new knowledge. Researchers are a subset of R&D personnel.

As with any dataset covering multiple countries and variables, there are vagaries. Brunei Darussalam, Columbia, Costa Rica, and Cote d'Ivoire report only Researcher FTE, however, their total FTE reported is less than 0.1% of the total FTE for all countries in our data set. We have imputed R&D personnel data from researcher FTE or count data, when available. As noted above in the Methods section, UNESCO R&D human resource data for the US is an extrapolation from a 20‐year‐old baseline.

Given these definitions of R&D personnel and researchers, we examined the relationship between those measures and those with a tertiary education. We hypothesize that researchers will be a subset of those with tertiary education. Figure 2 shows total R&D FTEs using the UNESCO data for all countries in our study dataset. We include, for context, researcher FTEs (a subset of R&D FTEs), doctorate and all tertiary education counts. Not all R&D human resources have a doctorate, and some do not have a tertiary degree. The exact degree of overlap differs by country and in many cases is not evident in the data. R&D personnel are about 2% of all tertiary‐educated, whilst researchers are close to 60% of R&D personnel. With these relationships in mind, we now consider research human capital density.

#### 3.2.2 Visualizing research intensity and research human capital density

**FIGURE 3:**
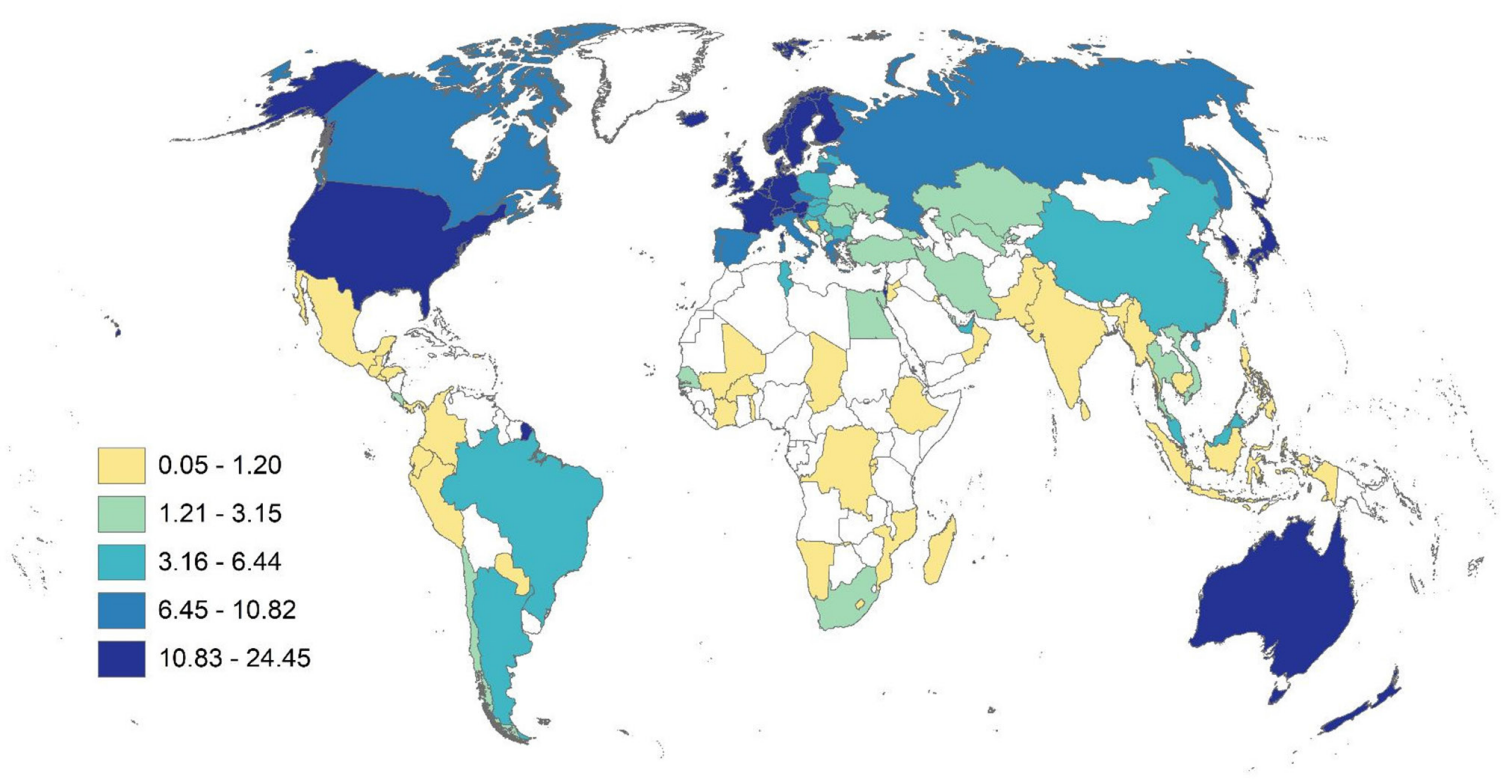
R&D personnel FTE, per 1000 population ages 25–69, Jenks distribution. Data derived from UNESCO and World Bank sources.

**FIGURE 4:**
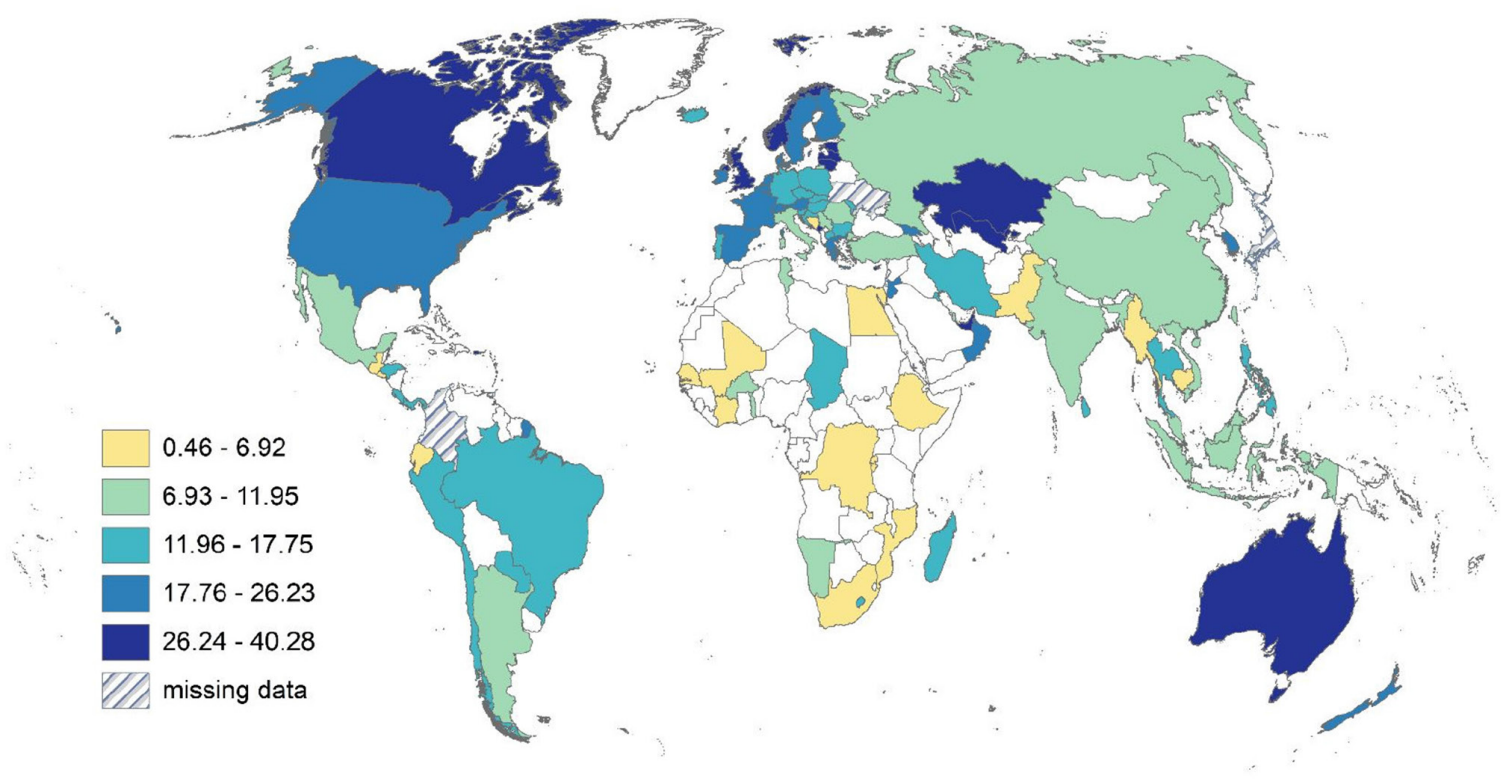
R&D person headcounts, per 1000 population ages 25–69, Jenks distribution. Data derived from UNESCO and NSCG sources.

**FIGURE 5:**
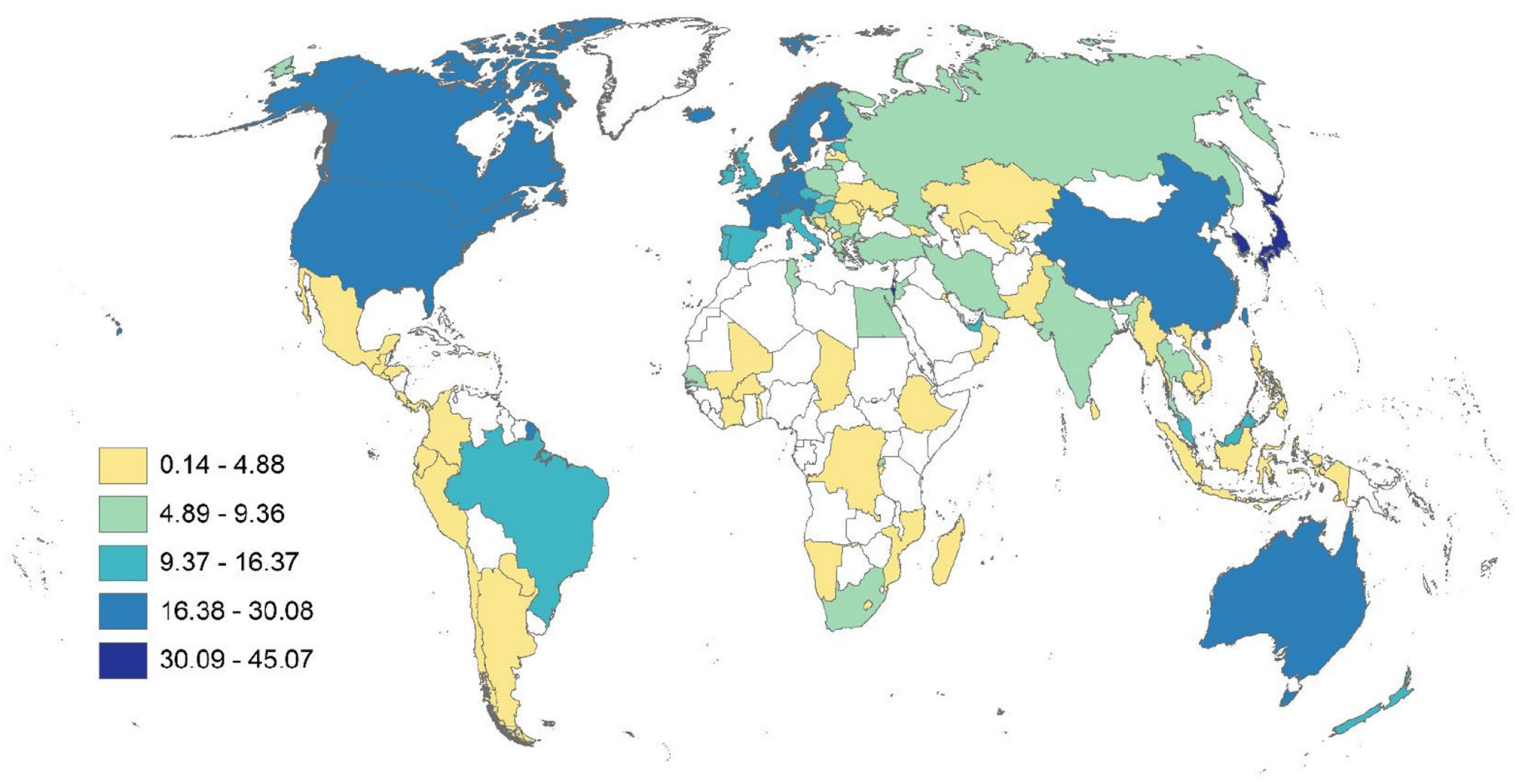
GERD as a proportion of GDP, per 1000 population ages 25–69, Jenks distribution. Data derived from UNESCO and World Bank sources.

We define research human capital density (RHCD) as the share of R&D FTE (or R&D persons) per 1000 population ages 25–69. Both OECD and UNESCO report measures of RHCD. The OECD measure is normalized per 1000 in the labour force (OECD Data, 2021), and the UNESCO definition is normalized per million population (UIS, 2021). Our measure is closer to the OECD measure and is normalized by the adult working population since definitions of labour force may vary across countries. Table 3 shows the headcount measures and RHCD by region. Whilst the North America and APAC regions have the highest numbers of R&D personnel and researchers, once these measures are normalized for population, EUS has the second highest RHCD. Because our objective is not just to count researchers, but also to assess differences between modes of measuring research human capital density, we mapped normalized data to the country level to ascertain qualitative differences between R&D FTEs (Figure 3) and R&D persons (Figure 4). We also include a map of the standard research intensity innovation metric R&D expenditures as a share of GDP (GERD) for comparison (Figure 5). There are clear differences between maps that may help to reshape our understanding of the innovation capacity of countries. In the supplement we include a map of R&D personnel headcounts, however, several countries, including North America, have missing data (Figure S1).

**TABLE 3:** Research and development human resources, by full‐time equivalent per 1000 capita, normalized to the total population from ages 24–69 years.

	R&D Personnel, FTE			Researchers, FTE			R&D Personnel, FTE per 1000 capita			Researchers, FTE per 1000 capita		
	N	Mean (Median)	SD	N	Mean (Median)	SD	N	Mean (Median)	SD	N	Mean (Median)	SD
APAC	18	346,322 (66,396)	903,788	19	188,981 (53,850)	401,211	19	4.86 (2.52)	5.02	18	3.66 (1.79)	3.97
ECA	11	98,310 (17,242)	236,328	11	56,778 (12,821)	126,487	11	2.93 (2.23)	2.23	11	1.94 (1.45)	1.19
EUS	31	100,965 (46,688)	152,499	31	64,400 (33,127)	94,863	31	9.93 (10.27)	4.62	31	6.59 (6.73)	3.21
LATAM	15	39,362 (3,208)	104.063	15	22,019 (1,642)	55,685	15	1.14 (1.12)	0.96	15	0.65 (0.57)	0.68
MEA	27	19,353 (2.588)	38,170	27	12,169 (1,162)	23,835	27	2.22 (0.63)	4.78	27	1.37 (0.38)	3.00
NA	2	1,199,364		2	769,859		2	11.29		2	7.45	

*Source*: UNESCO

First, examining modes of measurement, we see the highest research human capital densities in Australia, irrespective of mode. When comparing R&D FTEs to extrapolated R&D personnel headcounts, we see qualitative shifts in density, with relative regional increases in Latin America, Asia Pacific and Central Asia; mixed effects in African and North American countries; and decreases in Europe.

Comparing GERD to RHCD, we see different patterns. There is relatively more R&D spending compared with personnel in China, India, and Russia. In contrast, there are higher numbers of R&D personnel compared with spending in Australia and New Zealand; in Kazakhstan and Uzbekistan; across Europe; in Morocco; in Costa Rica, Chile and Argentina; and in the US.

These measures ‐ research intensity (GERD) and research human capital density (RHCD) ‐ present different aspects of a country's research capacity.

#### 3.2.3 Employer surveys compared to Self‐Reported R&D Activities

The UNESCO data are derived from employer‐based surveys and do not provide an individual perspective on research activities. Given the shift to a more inclusive definition of R&D in 2015, the employer‐based approach likely does not capture some types of research activities and hence may undercount people engaged in R&D activities.

To address these issues and explore other perspectives on R&D activities, we turned to the National Survey of College Graduates (NSCG, 2017), a biennial survey conducted by the US Census Bureau. The NSCG asks respondents whether they spend 10% of their time or more each week on basic research, applied research or development. Respondents are asked to choose their primary and secondary work activities. According to NSCG weighted tabulations in Table 4, there were 3.432 m US college graduates engaged in R&D (defined as a work activity that is basic research, applied research, or development) as a primary work activity, 6.054 m college graduates engaged in R&D as a secondary work activity in 2017, and an additional 11.28 m individuals who report spending at least 10% of their time at work on some aspect of R&D.

**TABLE 4:** US R&D human resources, 2014–2018.

Source	Variable	Effort	Headcounts	Weights	Estimated FTE
UNESCO UIS	US R&D Personnel FTE				2,163,950
US Census NSCG^a^	Counts, primary work activity is R&D	Full time	2,987,628	1	2,987,628
		Part time	444,636	0.5	222,318
	Counts, secondary work activity is R&D	Full time	5,118,129	0.25	1,279,532
		Part time	936,101	0.1	93,610
	People whose primary or secondary work activity is *not* R&D, BUT who report their work involves R&D	Full time	10,079,617	0.1	1,007,962
		Part time	1,202,259	0.05	60,113
	*Totals*		*20,768,370*		*5,651,163*

*Source*: NSCG (2017) and UNESCO (2014–2018).

^a^ Adjusted to remove indirect or ancillary activities, as specified in the Frascati definition.

We created an algorithm to convert NSCG headcount data to estimated FTE to enable a first‐pass comparison between NSCG and UNESCO R&D personnel figures and found a 2.6‐fold higher FTE count based on the NSCG data (Table 4). We assigned weights based on full‐time and part‐time employment, primary, secondary and any R&D work activity. Over 20 million people in the US report some R&D work activity and we estimate 5.65 million R&D FTE personnel.

Table 5 shows that over half of people indicating research as a primary work activity have a Bachelor's terminal degree, and two‐thirds are employed in the business sector. Engineering, Computer and Mathematical Sciences, General Management, Biological and Agriculture and Other Life Sciences, and Physical and Life Sciences are the top 5 occupations represented, making up 67.0% of the total. Writers, Editors, Press and Historians are also well‐represented, with 6.8% of the total. Clearly, R&D activities are performed by a broad spectrum of talent, across sectors and in a variety of occupations, although it should be noted that the NSCG does not capture those individuals performing research who do not have a college degree.

**TABLE 5:** US R&D Human Resources by Degree, Sector, and Occupation, 2017.

	R&D as Primary Work Activity	R&D as Primary or Secondary Work Activity
Degree		
Bachelors (ISCED 6)	59%	58%
Masters (ISCED 7)	27%	30%
Doctorate (ISCED 8)	12%	9%
Professional	2%	3%
Sector		
Education	17%	22%
Government	12%	11%
Business	71%	68%
Occupation		
Computer and Mathematical Sciences	14.4%	16.6%
Biological, Agriculture, and Other Life Sciences	8.1%	4.2%
Physical Sciences	7.1%	2.4%
Social Sciences	3.6%	3.3%
Engineering	25.0%	16.5%
Health Occupations	4.6%	7.3%
General Management	12.4%	15.1%
Teachers, K‐12	0.7%	6.2%
Teachers, Postsecondary	2.3%	3.5%
Social Work	2.2%	2.5%
Sales and Marketing	3.5%	7.2%
Writers, Editors, Historians, PR	6.8%	4.3%
Administrative Services	3.9%	3.5%
Professional Services	4.3%	4.0%
Construction, Precision Production, Maintenance, Transportation, and Other Occupations	3.9%	3.4%

*Source*: NSCG.

As a thought experiment, we extrapolated from NSCG data for personnel reporting R&D as a primary activity, to estimate global R&D personnel counts (Table 6). This requires many heroic assumptions and may in fact overestimate the number of researchers in the world. Nevertheless, it provides information above and beyond the R&D personnel numbers that in the US, may be a significant undercount. We first assume that the structure of the economy is similar in the US as in other countries. Second, we applied the weighting factor of US *R&D FTE / US tertiary education attainment* per capita, to each country's R&D FTE value (column 1, from UNESCO). Third, we normalized by tertiary education attainment per capita for each country (column 2). Fourth, since FTEs are fractions of total personnel counts; we further transformed the figures using country‐level FTE/headcount ratios from UNESCO data to obtain an estimate of R&D persons (column 3). Our extrapolation suggests that R&D FTE personnel in the world may be 62% greater than reported by UNESCO.

**TABLE 6:** Researcher FTEs and extrapolated FTEs and headcounts, by region and totals.

Region	R&D FTE (UNESCO)	Extrapolated R&D FTE	Extrapolated R&D Count
APAC	6,580,119	10,470,991	17,365,522
ECA	1,081,415	2,017,061	2,578,329
EUS	3,129,929	3,765,298	5,721,266
LATAM	590,423	1,807,345	2,938,128
MEA	522,539	1,235,678	2,166,905
NA	2,398,728	3,896,502	4,267,428
*Totals*	*14,303,153*	*23,192,875*	*35,037,579*

If we include in our estimates not only those who report R&D as a primary work activity but also those who report R&D as a secondary activity, we estimate that the global total rises to 97 million. These numbers are likely an overstatement since we are assuming: (1) that self‐reported R&D activity is the same as employer‐reported R&D activity; (2) that R&D production is the same in the US and all other countries; and (3) that the ratio of self‐reported R&D activity to tertiary education in the US would be the same in other countries. As we mentioned above, these are heroic assumptions. Nevertheless, the US data and this thought experiment suggest that we may be undercounting R&D personnel worldwide.

#### 3.2.4 Doctorates as R&D personnel

Another way of assessing innovation is to focus specifically on the doctorate population as is the focus of the National Science Board (NSB, 2020). We can examine the relationship between self‐reported R&D work activities, publication authorship and government research support. As seen in Figure 2, doctorates are a small share of R&D personnel. However, this population has been studied extensively because their work contributes to university and research rankings (ARWU, 2019; CWTS, 2020; National Science Board, 2020). Our goal is to examine whether authorship or research grants are proxies for being a researcher. For this analysis, we used the US National Science Foundation Survey of Doctoral Recipients (SDR) from 2015 (SDR, 2015). NSF has linked respondents in its 2015 SDR to Web of Science publications from 1990 to 2017. SDR respondents also report whether their work currently receives US Federal government research support (Table 7).

**TABLE 7:** Relationship between doctorate status, authorship, grants, and R&D occupation.

	Weighted total	Number with at least one publication	Number reporting support from US federal grants
Number of doctorates reporting primary work activity is R&D	364,337	311,579	130,327
Number of doctorates reporting secondary work activity is R&D	301,539	246,944	78,583
Total number of doctorates reporting employed status	787,250	732,439	216,328

*Source*: NSCG and SDR.

**FIGURE 6:**
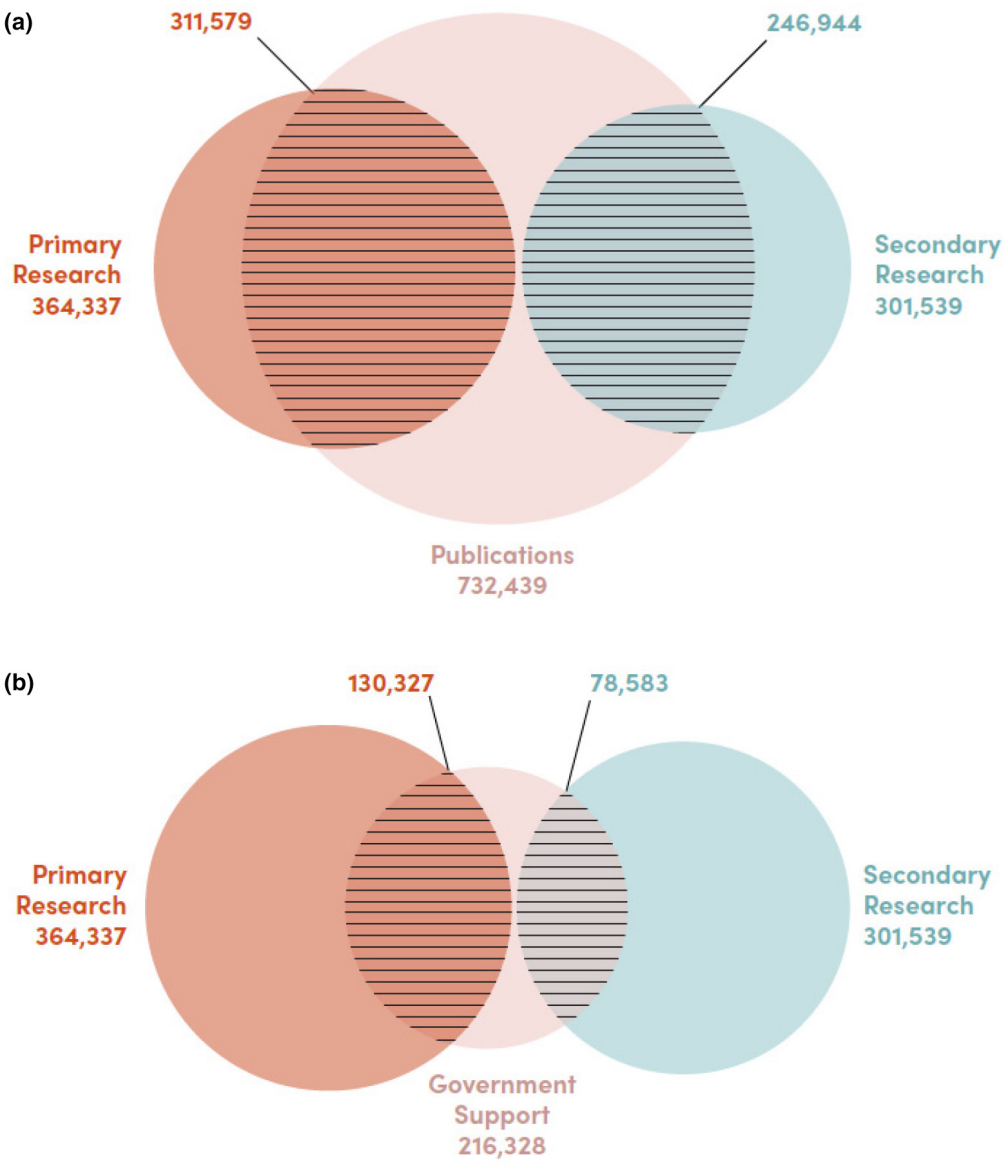
(a) Publications. Proportion of US Doctorates with at least one publication, 1990–2017. *Source*: 2015 NSF Survey of Doctorate Recipients matched to Web of Science publications. (b) Government Support. Proportion of US Doctorates with government research support. *Source*: NSF Survey of Doctorate Recipients, 2015.

Of the employed respondents, about 85% were in occupations that were either primary or secondary R&D focused. Furthermore, over 80% of doctorates employed in R&D occupations are linked to at least one publication, strongly suggesting that authorship can be used as a proxy for researcher status, at least amongst individuals with doctorate degrees. Figure 6a shows the overlap between primary work activity, secondary work activity and being an author on at least one publication. Only 14% of those reporting primary work as R&D and only 18% of those reporting secondary work as R&D do not have publications. It should be noted, however, that attempts to divide the number of unique authors based on the disambiguation of publication datasets are fraught by issues with name ambiguity (Kim, 2019), as well as low coverage of non‐English language journals and disciplinary variations in publication venues by field (Bello & Galindo‐Rueda, 2020).

Federal funding was less associated with reported research activity, with about 27% of employed doctorates reported having received any government research support in 2015. This may be misleading, as it excludes non‐federal awards from foundations, industry seed grants, and the like. Figure 6b shows the overlap between government support and primary and secondary R&D work activities. Government research support is a poor measure of R&D activities. Only 36% of those whose primary work activity and 26% of those whose secondary work activity are R&D have government support. This relatively weak relationship between research funding and research activities is instructive for how we measure overall research capacity.

### 3.3 Research production function estimates

What is the relative importance of research human capital density and research intensity in the production of research output measured by publications and patents? To explore this association, we estimated a series of multivariate regression models motivated by the production function literature where measures of innovated outputs are functions of the inputs of labour (RHCD) and capital (GERD).

Using our cross‐section of national data, we estimate models of the impact of RHCD and different types of GERD (total, business, and higher education) adjusted to a per capita measures on the outcomes. Our model also includes controls for per capita GDP, per capita tertiary educational enrollment and attainment, total ranked universities, as well as variables for governance and regional dummies (Table 8). These variables are included to control for cross‐country knowledge productive capacity. The number of ranked universities, tertiary educational enrollment and tertiary educational attainment are inputs in the production of publications and patents. Whilst tertiary enrollment can be considered a flow measure of tertiary attainment, it is measured at a point‐in‐time and is an input in the knowledge production process (Rosenbloom et al., 2015). The log of patent applications was regressed on the log of RHCD and the coefficient can be interpreted as an elasticity: a 1% increase in RHCD was associated with a 0.89 to 1% increase in patent applications depending on the measure of GERD used in the models. None of the estimates of GERD was associated with increased patenting. Additional top‐ranked universities were associated with more patents. We performed the same thought experiment with log publications. A 1% increase in RHCD was associated with an approximately 0.85% increase in publications. In the publication model, tertiary enrollment per capita was associated with increased publications as was GERD higher education funding. Interestingly, ranked universities were significantly associated with publications only when GERD higher education funding was used in the model.

**TABLE 8:** Patent application and publication volumes vs. economic and education variables.

Variables	(1)	(2)	(3)	(4)	(5)	(6)
	Log patent applications			Log publications		
Log RHCD, per capita	0.891*** **(0.108)**	0.997*** **(0.105)**	1.018*** **(0.096)**	0.846*** **(0.060)**	0.832*** **(0.053)**	0.852*** **(0.071)**
Log total tertiary enrollment, per capita	0.227 (0.362)	0.275 (0.359)	−0.060 (0.355)	0.656*** **(0.210)**	0.554** **(0.235)**	0.363 (0.238)
Log total tertiary attainment, per capita	0.422 (0.276)	0.443 (0.333)	0.636* **(0.322)**	−0.125 (0.143)	−0.179 (0.144)	−0.117 (0.185)
Log GERD, per capita	0.296 (0.245)			0.092 (0.132)		
Log GERD Higher Education, per capita		−0.073 (0.213)			0.180* **(0.095)**	
Log GERD Business Sector, per capita			0.027 (0.162)			0.051 (0.082)
Log GDP, per capita	−0.447 (0.461)	−0.155 (0.500)	−0.274 (0.389)	0.136 (0.257)	0.095 (0.220)	0.329 (0.307)
Total ranked universities	0.017*** **(0.003)**	0.017*** **(0.003)**	0.017*** **(0.003)**	0.002 (0.002)	0.003* **(0.002)**	0.003 (0.002)
Constant	−4.334 (4.327)	−7.300 (4.470)	−6.193 (4.453)	−4.354* **(2.511)**	−3.296 (2.316)	−4.853 (3.097)
Observations	88	85	79	99	96	80
*R*‐squared	0.900	0.896	0.906	0.943	0.946	0.940

*Note*: Each model includes governance variables (Government effectiveness, control of corruption, rule of law, political stability, voice and accountability, regularity quality) and region dummies. Robust standard errors are shown in parentheses. Items in bold are those values that meet statistical significance tests. ***p < 0.01, **p < 0.05, *p < 0.10.

We probed these results further in Table 9. In our first model, we include the log of GERD as a share of GDP, the measure of research intensity. In the second model, we include the log of RHCD, the measure of research human capital density, and in the last model, we include both. A 1% increase in research intensity (GERD) is associated with a 1.34% increase in patent applications (column 1) and a 0.82% increase in publications (column 4). A 1% increase in research human capital density (RHCD) is associated with 0.96% more patent applications (column 2) and 0.87% more publications (column 5). When we include both research intensity (GERD) and research human capital density (RHCD) in the models, the coefficients on GERD per capita drop in magnitude and are no longer statistically significant. However, research human capital density remains significantly associated with increases in both patents and publications. Thus, RHCD is more closely associated with research output than research expenditures measured by GERD.

**TABLE 9:** Patent application and publication volumes vs. economic and education variables.

Variables	(1)	(2)	(3)	(4)	(5)	(6)
	Log patent applications			Log publications		
Log of GERD, per capita	1.339*** **(0.254)**		0.301 (0.254)	0.815*** **(0.254)**		0.065 (0.129)
Log RHCD, per capita		0.960*** (0.087)	0.887*** (0.108)		0.869*** (0.052)	0.855*** (0.060)
Log total tertiary enrollment, per capita	−0.084 (0.411)	0.291 (0.323)	0.191 (0.353)	0.829** (0.352)	0.713*** (0.190)	0.704*** (0.201)
Log total tertiary attainment, per capita	0.899*** (0.273)	0.312 (0.278)	0.387 (0.264)	0.164 (0.260)	−0.087 (0.142)	−0.077 (0.148)
Total ranked universities	0.032** (0.006)	0.017*** (0.003)	0.017*** (0.003)	0.018 (0.005)	0.002 (0.002)	0.002 (0.002)
Constant	−1.647 (2.362)	−8.047** (2.288)	−7.583** (2.241)	2.240 (1.904)	−3.319** (1.041)	−3.250** (1.063)
Observations	88	88	88	99	99	99
R‐squared	0.789	0.897	0.900	0.792	0.942	0.942

*Note*: Each model includes governance variables (Government effectiveness, control of corruption, rule of law, political stability, voice and accountability, regularity quality) and region dummies. Robust standard errors are shown in parentheses. Items in bold are those values that meet statistical significance tests. ***p < 0.01, **p < 0.05, *p < 0.10.

## 4 DISCUSSION

In this paper, we have explored approaches to counting the number of researchers (R&D personnel) in the world using available data sources. We were inspired by the UNESCO operating definition of research and development, a broad definition that encompasses traditional knowledge, humanities and social sciences, product design, engineering and sciences. Indeed, R&D is not defined by field, occupation, or education, but rather, as an ‘activity [that is] novel, creative, uncertain in its outcome, systematic and transferable and/or reproducible’. We applied our findings to create national estimates of people doing R&D work, with a goal of providing a more holistic and inclusive appreciation of human capital that can drive capacity for innovation (Carayannis et al., 2018).

Using UNESCO data, we developed a measure of research human capital density that is the share of R&D personnel adjusted by population. However, this measure is based on employer surveys and may undercount the number of people engaged in R&D because it only provides the employer's perspective on R&D personnel and in the case of the US is extrapolated from a 20‐year‐old survey. Once we developed the RHCD measure, we compared the US R&D personnel measure to self‐reported research activities in the National Survey of College Graduates. Although the NSCG misses some people engaged in R&D who do not have college degrees, it does report the R&D work experiences of college graduates in the US workforce, providing information that connects R&D work activities with occupational data and tertiary educational attainment. The NSCG work activities information serves as a useful counterpoint to firm‐derived reports on R&D employment and could be used to develop country‐level measures of R&D (Beliaeva et al., 2018; Clauss et al., 2022).

Looking at the R&D workforce from the firm and individual perspectives yields substantially different numbers. UNESCO firm‐based surveys show about 14 m R&D FTEs in the world. However, when we used self‐reported measures of R&D activities from the NSCG data to extrapolate R&D personnel, our results show that the global count of researchers may be as high as 97 m. Whilst this extrapolation makes heroic assumptions, our results suggest that firm‐survey measures are likely undercounting global R&D human resource capability.

We examined the association between self‐reported R&D activities, research publications and government support of research, using the special case of US doctorates. We found considerable overlap in the share of doctorates with publications and reporting primary or secondary work activity as research. We found much less overlap in the share of doctorates reporting government support and primary or secondary work activity as research, about 9% of the US R&D workforce. Since doctorates with research funding are a small subset of total doctorates reporting research activities, this suggests that using research funding (GERD) as a measure of research intensity may understate the true amount of research taking place. Taken together, these results imply that authorship is a reasonable proxy for the subset of R&D personnel that are doctorates, and research funding will undercount research activities. These results also provide support for our argument that self‐reported R&D activity remains a primary avenue for measuring the R&D workforce. We recommend that organizations such as the US National Science Foundation and UNESCO adopt individual and occupational surveys to measure self‐reported research and development work activities for enhanced measures of R&D personnel. In addition, we recommend that self‐reported R&D activity measures be matched to firm‐level data to compare and contrast the efficacy of the two approaches.

We also tested whether research human capital density was associated with research output. We did so because many comparisons of global R&D use GERD as the preferred measure of research inputs (National Science Board, 2020). Our research production function framework underscores the importance of both capital (GERD) and labour (RHCD) in knowledge production. Our regression analysis demonstrates that an increase in RHCD is associated with significant increases in publications and patents. Furthermore, research intensity—GERD per capita—no longer has explanatory power after including measures of researcher density. Of course, these two measures are highly correlated (*ρ* = 0.9, *p* < 0.01) since GERD is likely supporting the wages and salaries of researchers. Nevertheless, the two measures are not the same. RHCD is significantly associated with publications and patents in our research production function models whilst the coefficient on GERD drops in magnitude and is no longer statistically significant. This suggests that countries that invest relatively more in R&D personnel produce more research output in the form of patents and publications. Our analysis does not explain why RHCD and GERD differ across countries. These differences will likely be driven by the size of the economy and geopolitical priorities in each country and are beyond the scope of this analysis.

Taken together, these findings suggest that comparisons of research intensity across countries may not adequately capture the R&D capacity of a country. GERD is convenient and straightforward to measure, but research output is dependent on research human capital density. Our results also show that the current UNESCO definitional separation of ‘researcher’ and ‘R&D person’ is not particularly useful and may underscore country and regional differences in data collection. Instead, we suggest equating ‘researcher’ and ‘R&D person’ to one term, defined as ‘any person that engages in or provides services to directly support R&D as a primary or secondary activity during their workday’. This term will include people who perform basic or applied research or experimental development in any knowledge field or sector.

UNESCO's open data collection of R&D variables, possibly along with innovative approaches to assessing the workforce (Berggren & Bjørnskov, 2021; Gomez et al., 2020; Martinelli et al., 2021), may support improved global coverage and more inclusive regional and worldwide studies, necessary for cross‐national work toward sustainable development goals, and we encourage UNESCO and other government bodies to highlight measures of research human capital density in their R&D reporting. The OECD reports researchers and R&D personnel per thousand in the labour force (OECD, 2021) and the UNESCO report researchers per million inhabitants (UIS, 2021).

That said, we suggest a shift in emphasis for cross‐national R&D comparisons. For example, the National Science Board's *Science and Engineering Indicators* chapter, *U.S. and Global Research and Development* only reports data on full‐time doctoral students in science and engineering as their measure of research human capital (National Science Board, 2020). As we have demonstrated, doctorates are a small share of total RHCD and RHCD is more predictive of R&D output measures reported in *Science and Engineering Indicators* than GERD.

Furthermore, we encourage countries that collect individual‐level and firm‐level data on R&D personnel to compare these measures as we did with US data. The ideal study would go further than our reported estimates. It would link individual‐self‐reported data to the firm that employs them and then compare the estimates of R&D activities. This kind of granular study will enhance our understanding of the R&D production process with the goal of more refined measures of ‘How many people in the world do research and development?’.

## FUNDING INFORMATION

This work was supported by the National Bureau of Economic Research and The Wellcome Trust, NBER Project No. [25410.00.00.00.6600] to LLH and the US National Science Foundation [SMA‐1854849] to DKG.

## Supporting information


Appendix S1.

## Data Availability

We used data from open sources with global reach and intercountry data quality standards and have created a study data set that is available for reuse (Haak, et al., 2021).

## Endnote

^1^
US R&D personnel data in the UNESCO database are extrapolated from data provided by the National Science Foundation Center on Science and Engineering Statistics (NCSES) to OECD about 20 years ago. At that time, The NCSES baseline totals for the Government sector were based on the US Office of Personnel Management (OPM) estimates for Federal scientists and engineers with R&D as their primary work activity. The NCSES estimates for the Education and Nonprofit sectors were estimated from the NSF Survey of Doctorate Recipients who reported R&D as their primary work activity. The NCSES is fielding new establishment surveys and data for business, government, education and non‐profit sectors will become available over the next 5 years.
