# Supplementary material for: How many people in the world do research and development?
Source: Glob Policy. 2023 Feb 8;14(2):270–87. doi: 10.1111/1758-5899.13182 (PMC10946615; doi:10.1111/1758-5899.13182)
Supplement: Supplementary file 1 — Appendix S1. [file GPOL-14-270-s001.docx]

How many people do research and development in the world?

Supplementary Figures and Tables

# Supplementary Table 1: Data Coverage Tables

| **Variable Name [Source]** | **APAC (19)** | **EUS (31)** | **ECA (11)** | **LATAM (15)** | **MEA (27)** | **NA (2)** | **Total (105)** |
| --- | --- | --- | --- | --- | --- | --- | --- |
| GDP [WDI] | 19 | 31 | 11 | 15 | 27 | 2 | 105 |
| GERD [UNESCO] | 19 | 31 | 11 | 15 | 27 | 2 | 105 |
| GERD - Business Sector [UNESCO] | 16 | 31 | 10 | 11 | 15 | 2 | 85 |
| GERD - Education Sector [UNESCO] | 19 | 31 | 11 | 14 | 25 | 2 | 102 |

**Table S1.a.** Countries in the dataset with investment data coverage for at least one year 2014-2018. Data sources are indicated in [square brackets]. Total country count per region is indicated in (parentheses).

| **Variable Name [Source]** | **APAC (19)** | **EUS (31)** | **ECA (11)** | **LATAM (15)** | **MEA (27)** | **NA (2)** | **Total (105)** |
| --- | --- | --- | --- | --- | --- | --- | --- |
| Enrollment ISCED 5  [UNESCO] | 19 | 26 | 6 | 12 | 23 | 2 | 88 |
| Enrollment ISCED 6  [UNESCO] | 19 | 31 | 10 | 12 | 27 | 2 | 101 |
| Enrollment ISCED 7  [UNESCO] | 19 | 31 | 10 | 12 | 27 | 2 | 101 |
| Enrollment ISCED 8  [UNESCO] | 19 | 31 | 11 | 13 | 27 | 2 | 103 |
| Total Tertiary Enrollment ISCED 5-8 [UNESCO] | 19 | 31 | 11 | 13 | 27 | 2 | 103 |
| Attainment ISCED 5-8 {imputed} [UNESCO] | 11 {18} | 29 {31} | 7 {10} | 12 {14} | 15 {27} | 2 | 76{102} |
| Attainment ISCED 6-8 {imputed} [UNESCO] | 9 {18} | 26 {30} | 6 {8} | 11 {14} | 15 {27} | 2 | 69{98} |
| Attainment ISCED 7-8 {imputed} [UNESCO] | 10 {18} | 25 {31} | 6 {10} | 10 {14} | 15 {27} | 2 | 68{102} |
| Attainment ISCED 8  {imputed} [UNESCO] | 9 {18} | 25 {31} | 5 {10} | 6 {14} | 14 {27} | 2 | 61{102} |

**Table S1.b**. Countries in the dataset with education data coverage for at least one year 2014-2018. Data sources are indicated in [square brackets]. Total country count per region is indicated in (parentheses). Imputed data are indicated by {curly brackets}.

| **Variable Name [Source]** | **APAC (19)** | **EUS (31)** | **ECA (11)** | **LATAM (15)** | **MEA (27)** | **NA (2)** | **Total (105)** |
| --- | --- | --- | --- | --- | --- | --- | --- |
| Total Employment [ILO] | 19 | 31 | 11 | 15 | 26 | 2 | 104 |
| Total Employment - Education Sector [UNESCO] | 4 | 26 | 2 | 5 | 1 | 0 | 38 |
| R&D FTE - Total [UNESCO] | 17 {19) | 31 | 11 | 11 {15} | 23 {27} | 1 {1} | 94{105} |
| R&D FTE - Business Sector [UNESCO] | 16 | 31 | 9 | 7 | 16 | 2 | 81 |
| R&D FTE - Education Sector [UNESCO] | 18 | 31 | 10 | 10 | 23 | 1 | 93 |
| R&D FTE - Government Sector [UNESCO] | 17 | 31 | 10 | 10 | 22 | 1 | 91 |
| Researcher FTE [UNESCO] | 17{19} | 31 | 11 | 13{15} | 23{27} | 2 | 97{105} |
| Technician FTE [UNESCO] | 16{17} | 20 | 11 | 11 | 23 | 1 | 82{83} |
| R&D Counts - Total [UNESCO] | 17 {19} | 31 | 11 | 14 | 26 | 0 {2} | 99{101} |
| R&D Counts - Business Sector [UNESCO] | 14 | 31 | 10 | 7 | 18 | 1 | 81 |
| R&D Counts - Education Sector [UNESCO] | 17 | 31 | 11 | 13 | 25 | 0 | 97 |
| R&D Counts - Government Sector [UNESCO] | 15 | 31 | 11 | 13 | 25 | 0 | 95 |

**Table S1.c**. Employment and researcher data coverage for at least one year 2014-2018. Data sources are indicated in [square brackets]. Total country count per region is indicated in (parentheses). Imputed data are indicated by {curly brackets}.

| **Variable Name [Source]** | **APAC (19)** | **EUS (31)** | **ECA (11)** | **LATAM (15)** | **MEA (27)** | **NA (2)** | **Total (105)** |
| --- | --- | --- | --- | --- | --- | --- | --- |
| Patent applications - residents [WIPO] | 18 | 31 | 10 | 13 | 17 | 2 | 91 |
| Research publications  [NSF] | 17 | 31 | 11 | 15 | 27 | 2 | 103 |
| Ranked University in Country >0  [Shanghai or Leiden] | 13 | 29 | 3 | 5 | 10 | 2 | 62 |
| Total Ranked Universities in Region  [Shanghai or Leiden] | 429 | 394 | 44 | 45 | 69 | 236 | 1217 |

**Table S1.d.** Ranked universities and patent and publication data coverage in 2019. Data sources are indicated in [square brackets]. Total country count per region is indicated in (parentheses).

| **Variable Name [Source]** | **APAC (19)** | **EUS (31)** | **ECA (11)** | **LATAM (15)** | **MEA (27)** | **NA (2)** | **Total (105)** |
| --- | --- | --- | --- | --- | --- | --- | --- |
| Government Effectiveness [WGI] | 19 | 31 | 11 | 15 | 27 | 2 | 105 |
| Control of Corruption  [WGI] | 19 | 31 | 11 | 15 | 27 | 2 | 105 |
| Political Stability [WGI] | 19 | 31 | 11 | 15 | 27 | 2 | 105 |
| Rule of Law [WGI] | 19 | 31 | 11 | 15 | 27 | 2 | 105 |
| Voice and Accountability [WGI] | 19 | 31 | 11 | 15 | 27 | 2 | 105 |
| Regulatory Quality [WGI] | 19 | 31 | 11 | 15 | 27 | 2 | 105 |

**Table S1.e.** Governance data coverage for at least one year 2014-2018. Data sources are indicated in [square brackets]. Total country count per region is indicated in (parentheses).

# Supplementary Table 2: Distribution of Data

We collected statistics from sources as described in the Methods section, averaged by region (**Tables S2.a-g**) over the time period 2014-2018. The sample showed variation by region in the income groups represented.

We examined patent applications and research publication volume, as well as the number of ranked universities, by country, for the time period 2014-2018 (**Table A2.b).** There is substantial variation in each variable by region. APAC, EUS, and NA have a 10-fold higher count of ranked universities. NA and APAC had 30-fold higher patent volumes than other regions but overall there was not a significant regional variation (ANOVA, p=0.233). NA publication volume was 5-80 times higher than other regions, and EUS had a moderately higher count of publications but not patents; overall there was a significant regional variation in publication outputs (ANOVA, p=0.0176**). Each of these variables is positively skewed, evident in the difference between the mean and median, as well as the large standard deviation. For correlation and regression analysis, we log-transformed patent and publication variables, and binned the university counts.

We examined economic factors that may influence innovation. In addition to total country-level GDP, we collected data on gross expenditures on research and development (GERD), as well as sub-categories of GERD for the business and education sectors (**Table A2.c**). Regional data were, for the most part, normally distributed, and showed 2-10 fold differences between regions (excluding NA). Overall, there was highly significant variation between regions for all GERD variables (ANOVA, p<0.0001, ***). We used per capita normalized variables for correlations and regressions.

Human resources play an important role in innovation. We collected data on people employed in research and development occupations (**Table A2.d**), as well as people enrolled in tertiary education (**Table A2.e**) or with higher education degrees (**Table A2.f)** -- groups that include R&D personnel and potential to engage in R&D activities. While absolute personnel FTEs varied substantially between countries and regions, this variation was reduced when normalized by population. This effect was more pronounced for tertiary educational enrollment and attainment variables. A number of countries in our sample were missing data for attainment during the 2014-2018 timeframe, and we imputed values based on available education data for these countries from 2010-2013. Overall, there was significant regional variation in R&D personnel (ANOVA, p=0.0254, **) and researcher variables (ANOVA, p= 0.411, **), and highly significant variability for all education variables (ANOVA, p<0.001, ***) We used population- normalized variables and imputed attainment variables for correlations and regressions.

Governance may also impact research and development activities. We collected country-level governance scores for each country, averaged the scores across the time period 2014-2018, and created regional summary statistics (**Table A2.g)**. The scores varied significantly across regions (ANOVA, p<0.0001, ***). We used average scores in correlation and regression analyses.

| ***Variable →*** | **N** | **Income Group [UNESCO]** | | | | **Employment, per capita [ILO]** | |
| --- | --- | --- | --- | --- | --- | --- | --- |
| ***Region*** |  | **High** | **Upper Middle** | **Lower Middle** | **Low** | **Mean**  **(Median)** | **SD** |
| **APAC** | 19 | 8 | 4 | 7 | 0 | 0.87  (0.88) | 0.12 |
| **ECA** | 11 | 0 | 8 | 3 | 0 | 0.72  (0.77) | 0.13 |
| **EUS** | 31 | 30 | 1 | 0 | 0 | 0.77  (0.76) | 0.09 |
| **LATAM** | 15 | 4 | 2 | 9 | 0 | 0.87  (0.88) | 0.14 |
| **MEA** | 27 | 8 | 4 | 5 | 10 | 0.91  (0.87) | 0.26 |
| **NA** | 2 | 2 | 0 | 0 | 0 | 0.85 |  |

**Table S2.a.** Income distribution of countries in sample, by region. SOURCE: UNESCO and ILO

|  | **Patent applications, residents [WIPO]** | | | **Number of Scientific and technical journal articles [NSF]** | | | **Ranked Universities [Shanghai, CWTS]** | | | | |
| --- | --- | --- | --- | --- | --- | --- | --- | --- | --- | --- | --- |
|  | **N** | **Mean (Median)** | **SD** | **N** | **Mean (Median)** | **SD** | **Region**  **Total** | **Ave (SD)** | **0** | **1-4** | **5+** |
| **APAC** | 18 | 87,224 (1,094) | 267,675 | 17 | 50,419  (10,094) | 108,468 | 429 | 22.6 (52.0) | 6 | 4 | 9 |
| **ECA** | 10 | 3,614  (272) | 7,958 | 11 | 10,288  (610) | 19,854 | 44 | 4 (9.24) | 8 | 1 | 2 |
| **EUS** | 31 | 3,517  (811) | 8,977 | 31 | 21,491  (11.173) | 28,754 | 394 | 12.7 (17.0) | 2 | 10 | 19 |
| **LATAM** | 13 | 612  (53) | 1,372 | 15 | 6,420  (583) | 14,361 | 45 | 3 (7.69) | 10 | 2 | 3 |
| **MEA** | 17 | 1,016  (16) | 3,355 | 27 | 3,456  (223) | 8,594 | 69 | 2.6 (7.12) | 17 | 6 | 4 |
| **NA** | 2 | 146,871 |  | 2 | 244,701 |  | 236 | 118 | 0 | 0 | 2 |

**Table S2.b.** Patent application, publications, and ranked university counts, by region. SOURCES: WIPO, NSF, Shanghai, and CWTS.

|  | **GERD, per capita** | | | **GERD % of GDP** | | | **GERD Business Sector,**  **per capita** | | | **GERD Education Sector,**  **per capita** | | |
| --- | --- | --- | --- | --- | --- | --- | --- | --- | --- | --- | --- | --- |
|  | **N** | **Mean**  **(Median)** | **SD** | **N** | **Mean**  **(Median)** | **SD** | **N** | **Mean**  **(median)** | **SD** | **N** | **Mean**  **(Median)** | **SD** |
| **APAC** | 19 | 576.7  (232.7) | 787.4 | 19 | 1.06  (0.68) | 1.17 | 16 | 422.9  (191.1) | 600.5 | 19 | 150.6  (30.05) | 188.2 |
| **ECA** | 11 | 101.1 (60.78) | 97.13 | 11 | 0.47  (0.36) | 0.33 | 10 | 48.46  (19.27) | 58.89 | 11 | 31.42  (24.88) | 32.54 |
| **EUS** | 31 | 965.4  (790.2) | 699.9 | 31 | 1.68  (1.36) | 0.88 | 31 | 611.5  (467.2) | 493.4 | 31 | 250.3  (193.6) | 185.7 |
| **LATAM** | 15 | 88.66 (59.19) | 83.57 | 15 | 0.32  (0.27) | 0.31 | 11 | 33.86  (27.10) | 43.26 | 14 | 24.00  (13.71) | 22.62 |
| **MEA** | 27 | 223.4  (72.76) | 557.6 | 27 | 0.55  (0.35) | 0.84 | 15 | 224.0  (14.90) | 623.21 | 25 | 61.52  (13.77) | 125.12 |
| **NA** | 2 | 1,734 |  | 2 | 2.22 |  | 2 | 1,141 |  | 2 | 377.8 |  |

**Table S2.c.**  Gross expenditure in research and development (GERD), total and by sector. Per capita is normalized to the total population from ages 24-69 years. GDP is expressed as PPP, in constant 2017 international dollars. SOURCES: UNESCO (GERD) and WDI (GDP, population).

|  | **R&D Personnel, FTE** | | | **Researchers, FTE** | | | **R&D Personnel, FTE per 1000 capita** | | | **Researchers, FTE per 1000 capita** | | |
| --- | --- | --- | --- | --- | --- | --- | --- | --- | --- | --- | --- | --- |
|  | **N** | **Mean**  **(Median)** | **SD** | **N** | **Mean**  **(Median)** | **SD** | **N** | **Mean**  **(Median)** | **SD** | **N** | **Mean**  **(Median)** | **SD** |
| **APAC** | 18 | 346,322 (66,396) | 903,788 | 19 | 188,981 (53,850) | 401,211 | 19 | 4.86  (2.52) | 5.02 | 18 | 3.66  (1.79) | 3.97 |
| **ECA** | 11 | 98,310  (17,242) | 236,328 | 11 | 56,778  (12,821) | 126,487 | 11 | 2.93  (2.23) | 2.23 | 11 | 1.94 (1.45) | 1.19 |
| **EUS** | 31 | 100,965 (46,688) | 152,499 | 31 | 64,400  (33,127) | 94,863 | 31 | 9.93  (10.27) | 4.62 | 31 | 6.59 (6.73) | 3.21 |
| **LATAM** | 15 | 39,362  (3,208) | 104.063 | 15 | 22,019  (1,642) | 55,685 | 15 | 1.14  (1.12) | 0.96 | 15 | 0.65 (0.57) | 0.68 |
| **MEA** | 27 | 19,353  (2.588) | 38,170 | 27 | 12,169  (1,162) | 23,835 | 27 | 2.22  (0.63) | 4.78 | 27 | 1.37 (0.38) | 3.00 |
| **NA** | 2 | 1,199,364 |  | 2 | 769,859 |  | 2 | 11.29 |  | 2 | 7.45 |  |

**Table S2.d.**  Research and development human resources, by full-time equivalent per 1000 capita, normalized to the total population from ages 24-69 years. SOURCE: UNESCO

|  | **N** | **Total Count**  **(ISCED 5-8)** | | **Doctoral Count**  **(ISCED 8)** | | **Total per 1000 capita** | | **Doctorates per 1000 capita** | |
| --- | --- | --- | --- | --- | --- | --- | --- | --- | --- |
|  |  | **Mean (Median)** | **SD** | **Mean**  **(Median)** | **SD** | **Mean**  **(Median)** | **SD** | **Mean**  **(Median)** | **SD** |
| **APAC** | 19 | 5,592,964 (1,745,539) | 11,741,985 | 45,374 (21,160 ) | 79,645 | 59.46  (51.88) | 26.93 | 1.23  (0.59) | 1.35 |
| **ECU** | 11 | 1,478,984 (250,669) | 2,504,964 | 22,484 (2,511) | 39,040 | 65.02  (62.42) | 33.41 | 0.95  (1.06) | 0.80 |
| **EUS** | 31 | 654,240 (300,576) | 834,117 | 25,309 (14,835 ) | 40,244 | 67.97  (65.53) | 18.54 | 2.58  (2.41) | 1.28 |
| **LATAM** | 13 | 1,770,855 (705,498) | 2,360,030 | 14,761 (1,012) | 29,772 | 87.74  (79.94) | 27.61 | 0.44  (0.22) | 0.43 |
| **MEA** | 27 | 445,523 (123,587) | 938,080 | 8,609 (1,002) | 23,728 | 35.55  24.82) | 23.32 | 0.51  (0.15) | 0.75 |
| **NA** | 2 | 10,446,042 |  | 215,383 |  | 88.83 |  | 2.23 |  |

**Table S2.e.**  Tertiary education enrollment, by region. Total includes 2-year (ISCED 5) and 4-year post-secondary degrees (ISCED 6), Masters degrees (ISCED 7), and doctoral degrees (ISCED 8), as defined in the Frascati Manual. SOURCE: UNESCO.

|  | **Total Count**  **(ISCED 5-8)** | | | **Doctoral Count**  **(ISCED 8)** | | | **Total per 1000 capita** | | | **Doctorates per 1000 capita** | | |
| --- | --- | --- | --- | --- | --- | --- | --- | --- | --- | --- | --- | --- |
|  | **N** | **Mean**  **(Median)** | **SD** | **N** | **Mean**  **(Median)** | **SD** | **N** | **Mean**  **(Median)** | **SD** | **N** | **Mean**  **(Median)** | **SD** |
| **APAC** | 18 (7) | 15,998,016 (5,429,231) | 32,612,114 | 18 (9) | 132,758 (33,701) | 206,520 | 18 (7) | 228.7  (189.9) | 133.5 | 18 (9) | 3.14  (1.36) | 3.70 |
| **ECA** | 10 (3) | 5,547,149 (932,607) | 8,491,168 | 10 (5) | 55,856  (10,185) | 100,118 | 10 (3) | 341.1  (269.9) | 206.1 | 10 (5) | 2.59  (2.01) | 1.62 |
| **EUS** | 31 (2) | 3,340,324 (1,447,725) | 4,800,708 | 31 (6) | 95,021 (40,462) | 167,384 | 31 (2) | 338.5  (354.5) | 91.70 | 31 (6) | 8.84  (7.23) | 7.14 |
| **LATAM** | 14 (2) | 3,550,291 (793,052) | 5,290,920 | 14 (8) | 44,595 (15,428) | 75,957 | 14 (2) | 182.5  (179.8) | 69.88 | 14 (8) | 2.65  (0.99) | 2.78 |
| **MEA** | 27 (12) | 1,258,614 (445,718) | 2,377,282 | 27 (13) | 28,008 (9,009) | 41,519 | 27 (12) | 146.6  (143.8) | 124.0 | 27 (13) | 2.96 (2.24) | 3.67 |
| **NA** | 2 (0) | 53,579,142 |  | 2 (0) | 2,129,364 |  | 2 (0) | 547.8 |  | 2 (0) | 16.49 |  |

**Table S2.f.**  Tertiary education attainment, by region. Total includes 2-year (ISCED 5) and 4-year post-secondary degrees (ISCED 6), Masters degrees (ISCED 7), and doctoral degrees (ISCED 8), as defined in the Frascati Manual. Values imputed from earlier years (2010-2014) for the number of countries shown in parentheses in the *N* column. SOURCE: UNESCO

|  | **N** | **Government Effectiveness** | | **Control of Corruption** | | **Political Stability, Absence of Violence** | | **Regulatory Quality** | | **Rule of Law** | | **Voice and Accountability** | |
| --- | --- | --- | --- | --- | --- | --- | --- | --- | --- | --- | --- | --- | --- |
|  |  | **Mean (Med)** | **SD** | **Mean (Med)** | **SD** | **Mean (Med)** | **SD** | **Mean (Med)** | **SD** | **Mean (Med)** | **SD** | **Mean (Med)** | **SD** |
| **APAC** | 19 | 0.64 (0.40) | 0.98 | 0.32 (-0.28) | 1.08 | 0.09  (0.14) | 1.07 | 0.56 (0.20) | 1.06 | 0.42 (0.01) | 1.01 | -0.14 (-0.13) | 0.91 |
| **ECU** | 11 | -0.11 (0.03) | 0.37 | -0.47 (-0.45) | 0.53 | -0.50 (-0.30) | 0.67 | -0.06 (-0.02) | 0.63 | -0.36 (-0.23) | 0.40 | -0.41 (-0.14) | 0.68 |
| **EUS** | 31 | 1.17 (1.10) | 0.56 | 1.10 (0.91) | 0.81 | 0.75  (0.77) | 0.39 | 1.21 (1.17) | 0.49 | 1.20 (1.13) | 0.63 | 1.14 (1.18) | 0.37 |
| **LATAM** | 13 | -0.09 (-0.06) | 0.47 | -0.31 (-0.46) | 0.57 | -0.09 (-0.08) | 0.48 | 0.12 (0.10) | 0.60 | -0.27 (-0.50) | 0.64 | 0.27 (0.24) | 0.45 |
| **MEA** | 27 | -0.25 (-0.29) | 0.82 | -0.17 (-0.08) | 0.70 | -0.51 (-0.51) | 0.87 | -0.22 (-0.38) | 0.72 | -0.20 (-0.14) | 0.70 | -0.52 (-0.63) | 0.75 |
| **NA** | 2 | 1.64 |  | 1.63 |  | 0.83 |  | 1.62 |  | 1.71 |  | 1.27 |  |

**Table S2.g.**  Country-level governance indicators, average score over 2014-2018. SOURCE: WGI.

#

# Supplementary Figure 1: Reported R&D Headcounts

In addition to mapping RD FTE and extrapolated headcount data, we also mapped headcount data from UNESCO data. As figure S.5. shows, in addition to several countries with missing data (US, Canada, Columbia, India, Australia), we see qualitative differences across regions, with higher relative headcounts in Latin America and APAC countries, and mixed differences in Europe and Central Asia.
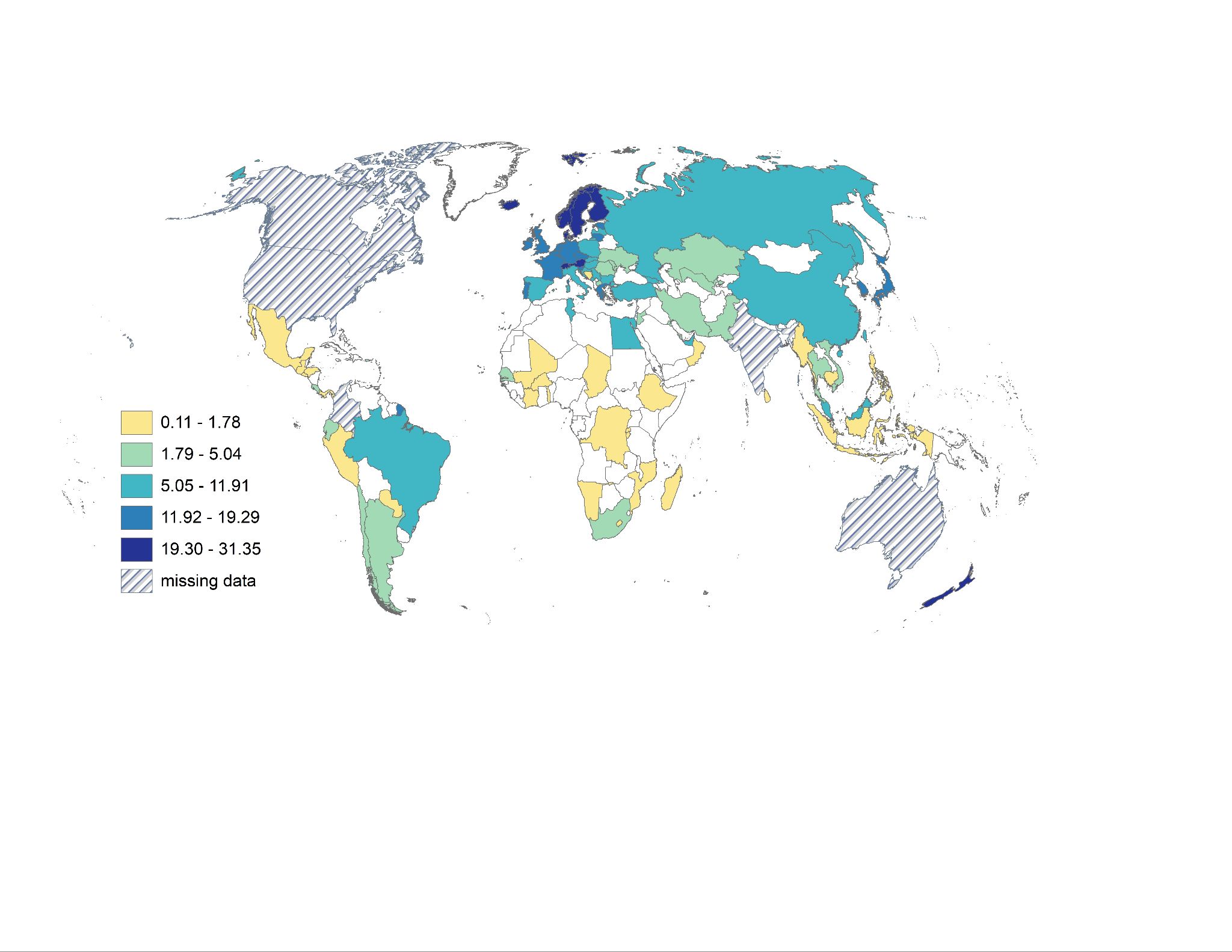


**Figure S.1.**  R&D person headcounts, per 1000 population ages 25-69, Jenks distribution. SOURCE: UNESCO.
